# Factors Influencing Medical Interns' Volunteering at a United Arab Emirates (UAE) Tertiary Care Institution

**DOI:** 10.7759/cureus.112166

**Published:** 2026-07-06

**Authors:** Isra Abbas, Zeinab Al-Rawi, Kaltham AlShaibah, Behishta Wazirzai, Sofia Alfalasi, Maryam Salman, Maitha Aljuwaied, Ayesha Almheiri

**Affiliations:** 1 Medicine, Mohammed Bin Rashid University of Medicine and Health Sciences, Dubai, ARE; 2 Family Medicine, Mohammed Bin Rashid University of Medicine and Health Sciences, Dubai, ARE

**Keywords:** challenges in volunteering, healthcare training, medical interns, medical internship, post graduation medical education, uae healthcare, volunteering activities

## Abstract

Background

Volunteering among medical interns is an understudied area with implications for personal, professional, and community development. The motivations and barriers influencing intern participation vary and remain insufficiently characterised, particularly within the Gulf Cooperation Council (GCC) region. This study addresses this gap by examining factors that motivate and hinder volunteering among medical interns at Dubai Health.

Methods

A cross-sectional survey was conducted from January to February 2024. Data were collected via an online self-administered questionnaire adapted from a validated tool. The target population comprised medical interns at Dubai Health enrolled during the cohort period of August 2023 to August 2024. A sample size of 63 was calculated using a 95% confidence level, a 5% margin of error, and an anticipated response rate of 90%. Data were analysed using Excel (Microsoft® Corp., Redmond, WA).

Results

The survey was completed by 65 medical interns, of whom 55 (84.6%) reported prior volunteering experience and 10 (15.4%) had no prior involvement. Among those with prior experience, 85.5% (n=47) had engaged in medical volunteering and 52.7% (n=29) in non-medical activities; some interns participated in both. The most frequently cited motivations among volunteers (n=55) were serving the community (92.7%, n=51), seeking experiences for residency applications or career development (67.3%, n=37), and skill enhancement (63.6%, n=35). Among the 10 non-volunteers, the principal barriers were heavy workload or time constraints and lack of awareness of volunteering opportunities, each reported by 90.0% (n=9). As participants could select multiple options, percentages may exceed 100%.

Conclusion

Medical interns at Dubai Health demonstrate high rates of volunteering, driven primarily by community service and career-related motivations. Among non-volunteers, workload and lack of awareness are the predominant barriers. Targeted institutional support and improved communication about available opportunities may increase participation.

## Introduction

Volunteering involves individuals offering their time and skills to benefit others, particularly within the community. It may be formal or informal and is often driven by aspirations for both personal and professional growth [1]. For medical interns, volunteering provides opportunities to develop essential clinical and interpersonal skills, make meaningful contributions to society, and strengthen their professional profiles. Such activities may occur in diverse settings, including community centres, sports facilities, rehabilitation shelters, and healthcare institutions, offering valuable hands-on experience [2].

A growing body of literature has assessed volunteering motivations and barriers among undergraduate medical students and interns across diverse settings, including within the Gulf Cooperation Council (GCC) countries. Studies among medical students in Saudi Arabia reported high motivation to volunteer, with participants citing benefits such as skill development, community contribution, and CV enhancement, despite institutional barriers such as academic commitments [3]. In the United Arab Emirates specifically, Al Saraidi et al. assessed motivations and barriers among healthcare volunteers using a functional-motives framework, finding that understanding and self-esteem were the most prominent motivating dimensions, while barriers spanned individual factors, such as work and family commitments, and organisational factors, such as insufficient information about volunteering opportunities [4]. Volunteerism frameworks describe a range of motives, such as protection, values, social connections, understanding, career development, personal growth, self-esteem, reciprocity, recognition, and reactivity. Nonetheless, barriers persist, including limited time, mobility restrictions, health concerns, and legal or regulatory limitations. Predictors of willingness to volunteer include individual characteristics, available resources, and social influences [5,6].

In the United Arab Emirates, medical interns can engage in volunteering through structured programs coordinated by healthcare institutions, government-led initiatives, and community-based organisations [7]. These programs follow established procedures that ensure compliance with professional, ethical, and regulatory standards. Although several studies have explored volunteering motivations and challenges among healthcare students in the United Arab Emirates [4], few have focused specifically on medical interns, whose roles and constraints differ from those of other student groups. This study aims to address this gap by examining the factors that motivate and hinder volunteering among medical interns at Dubai Health, providing insights to guide future initiatives and policy development.

## Materials and methods

Study design

This was a cross-sectional survey conducted over a two-month period from January to February 2024. Ethical approval was obtained from the University Student and Resident Research Subcommittee (USRRSC) of the Mohammed Bin Rashid University of Medicine and Health Sciences Institutional Review Board (MBRU-IRB), Dubai, United Arab Emirates. At Dubai Health, medical interns may participate in volunteering activities through institutionally coordinated programmes, which operate alongside their clinical duties and are subject to professional and ethical guidelines set by the organisation.

Study population and sample size

The target population comprised all medical interns at Dubai Health enrolled during the internship cohort period of August 2023 to August 2024. The survey was administered during a two-month window within this period, from January to February 2024. Based on institutional records, the total number of interns during this cohort period was approximately 70. The sample size was calculated using the Raosoft online sample size calculator with a 95% confidence level, a 5% margin of error, and an anticipated response rate of 90%, yielding a minimum required sample size of 63 participants [8], which was deemed sufficient to ensure representativeness and statistical reliability within the scope of this single-institution study. Interns were invited to participate via institutional emails.

Data collection

Data were collected through an online self-administered survey adapted from a previously validated tool by Al Saraidi et al. [4]. The survey is attached in Appendix 1. The survey was divided into multiple sections. Participants were initially questioned about their previous volunteering experience. Those with prior volunteering experience were directed to questions regarding the type of activities they had been involved in, whether medical or non-medical, and the factors that motivated them to volunteer, such as serving the community, skill enhancement, networking opportunities, mental health benefits, and influence from peers or faculty. Participants without prior volunteering experience were redirected to questions exploring the barriers that prevented them from volunteering, including heavy workload, lack of interest, transport issues, and health concerns. The survey was structured using closed-ended questions to facilitate quantitative analysis and enable comparability with previous studies; this format was also chosen to maintain brevity and encourage participation, given the time constraints inherent to medical internship duties.

Data analysis

Data were entered and analysed using Excel (Microsoft® Corp., Redmond, WA). Responses were summarised using frequencies and percentages, and results are presented in tabular form. Motivating factors were analysed among volunteers (n=55), while barriers were analysed among non-volunteers (n=10), as each group completed only the relevant section of the survey. As multiple responses were permitted for each item, percentages represent the proportion of respondents within each group selecting each factor and therefore do not sum to 100%.

## Results

The survey was completed by 65 medical interns, of whom 55 (84.6%) reported prior volunteering experience and 10 (15.4%) had no prior involvement. Among those with prior volunteering experience, 85.5% (n=47) had engaged in medical-related activities and 52.7% (n=29) in non-medical volunteer work; some interns participated in both, resulting in overlapping percentages. A summary of all responses is presented in Table 1.

**Table 1:** Volunteering Experience, Motivating Factors, and Barriers Among Medical Interns at Dubai Health (N = 65)

Question	Option	N	%
Q1: Previous Volunteering Experience (N=65)	Yes	55	84.6%
	No	10	15.4%
Q2: Type of Volunteering Activities (n=55, multiple selections permitted)	Medical	47	85.5%
	Non-medical	29	52.7%
Q3: Motivating Factors (n=55, multiple selections permitted)	Serving the community	51	92.7%
	Seeking experiences for residency/career	37	67.3%
	Skill enhancement	35	63.6%
	Networking/Building connections	27	49.1%
	Peers/faculty influence	24	43.6%
	Seeking a better understanding of the challenges faced by others	23	41.8%
	Religious consideration/Good deed	21	38.2%
	Reducing stress/Enhancing mental health	19	34.5%
	Availability of sufficient time	14	25.5%
	Reciprocation/Good Karma	11	20.0%
	Financial compensation	5	9.1%
Q4: Barriers to Volunteering (n=10, multiple selections permitted)	Heavy workload/Time constraints	9	90.0%
	Lack of awareness about opportunities	9	90.0%
	Distant location/Transport issues	3	30.0%
	Physical or mental health issues	1	10.0%
	Lack of interest	0	0%
	Financial constraints	0	0%
	Lack of legal or policy regulation	0	0%

The most frequently cited motivating factors among volunteers (n=55) were serving the community (92.7%, n=51), seeking experiences for residency applications and career development (67.3%, n=37), and skill enhancement (63.6%, n=35). Additional motivators included networking and building connections (49.1%, n=27), peer or faculty influence (43.6%, n=24), seeking a better understanding of challenges faced by others (41.8%, n=23), religious considerations or performing good deeds (38.2%, n=21), reducing stress or enhancing mental health (34.5%, n=19), availability of sufficient time (25.5%, n=14), reciprocation or good karma (20.0%, n=11), and financial compensation (9.1%, n=5). As participants could select multiple motivating factors, percentages reflect the proportion of volunteers endorsing each factor and may exceed 100%.

Among non-volunteers (n=10), the principal barriers were heavy workload or time constraints and lack of awareness about volunteering opportunities, each cited by 90.0% (n=9). Further barriers included distant location or transport issues (30.0%, n=3) and physical or mental health concerns (10.0%, n=1). No participants reported a lack of interest, financial constraints, or a lack of legal or policy regulation as barriers. As with motivating factors, participants could select multiple barriers; percentages therefore reflect the proportion of non-volunteers endorsing each barrier.

## Discussion

Our study found that the majority of medical interns at Dubai Health (84.6%) had prior volunteering experience, predominantly within the medical field. This is notably higher than rates reported among undergraduate medical students in prior research, where engagement was recorded at 60% among third-year students and 34% among final-year students [6].

Motivations for volunteering

The primary motivations among volunteers were serving the community (92.7%, n=51) and seeking experiences for residency applications or career development (67.3%, n=37). These findings align with previous studies highlighting personal interest and CV enhancement as key motivators for medical student volunteering [6]. Our results also support the volunteer functions inventory (VFI) model, which categorises motivations into six domains: Protective, Values, Social, Understanding, Career, and Enhancement. Career development motivations align with the Career and Enhancement domains, while the desire to serve the community reflects the Values and Protective domains, highlighting the multifaceted nature of volunteering [5,9].

Another framework that helps contextualise these findings is self-determination theory (SDT), which distinguishes between intrinsic and extrinsic motivation [10,11]. Intrinsic motivation encompasses the personal satisfaction and enjoyment derived from volunteering, while extrinsic motivation is driven by external rewards such as career advancement or networking opportunities. The prominence of both community service and career-related motivations in our findings reflects this dual dynamic, suggesting that intern volunteering is driven by a complex interplay of personal fulfilment and professional aspiration rather than a single motivational type.

Beyond these primary drivers, several secondary motivations warrant consideration. Skill enhancement was cited by 63.6% (n=35), reinforcing the role of volunteering as a platform for professional development. Peer and faculty influence (43.6%, n=24) also played a notable role, consistent with literature highlighting the importance of social networks and recruitment in volunteering decisions [12]. Religious considerations motivated 38.2% (n=21), aligned with research identifying religiosity as a positive predictor of volunteer participation [13,14]. Notably, stress reduction or mental health benefits were reported by 34.5% (n=19), suggesting that volunteering serves not only professional but also personal well-being needs among this cohort.

Barriers to volunteering

Several factors may hinder volunteering among interns, including time constraints, mobility challenges, transportation issues, health concerns, and lack of accessible information about volunteering opportunities [15,16]. These barriers align with previous studies identifying workload and time limitations as key obstacles to volunteering. In our study, heavy workload and time constraints were reported as the most significant deterrents, cited by 90.0% (n=9) of non-volunteering participants. Lack of awareness about volunteering opportunities was equally common (90.0%, n=9), emphasising the need for improved communication and organisation within healthcare institutions to better facilitate volunteerism. Similarly, logistical and structural barriers, such as a lack of information and formal policies governing volunteer activities, have been noted in earlier research [16]. Other barriers included transport difficulties (30.0%, n=3), while physical or mental health concerns (10.0%, n=1) are consistent with research identifying health status as a constraint on volunteering participation [17]. These findings highlight the importance of designing volunteering opportunities that are inclusive and accessible.

Notably, while heavy workload and time constraints were the predominant barrier among non-volunteers, 25.5% of volunteers (n=14) cited the availability of sufficient time as a motivating factor for their participation. As these findings come from two distinct groups, this contrast is descriptive rather than causal. Future research distinguishing structural time availability, such as rotation type or scheduling flexibility, from individual time management behaviour would help clarify whether institutional scheduling changes could meaningfully shift participation.

While our study was not conducted during a crisis period, research during the COVID-19 pandemic in Saudi Arabia found that logistical factors, such as workplace proximity, transportation, and institutional support, played a key role in determining volunteer willingness [18]. This is consistent with our findings, where logistical barriers such as transport difficulties and lack of awareness were among the barriers reported, highlighting that such challenges extend beyond crisis settings.

When interpreting these findings, it is important to consider both the strengths and limitations of the study design.

Study strengths

This study has several notable strengths. It is among the first to examine volunteering motivations and barriers specifically among medical interns at a UAE healthcare institution, addressing a gap in the existing literature. The study achieved a high response rate, with 65 of approximately 70 eligible interns participating (92.9%), enhancing the representativeness of findings within the study setting. Data were collected using a questionnaire adapted from a previously validated tool by Al Saraidi et al. [4], supporting the reliability of the instrument. Furthermore, the study provides a contextually relevant evidence base that can inform institutional policy and programme development for intern volunteering at Dubai Health and similar settings.

Study limitations

The sample size of 65 interns was calculated to meet the statistical requirements for this study; however, it remains relatively small compared to larger studies such as those by Pop et al. and Alslamah et al., which included 510 and 410 participants, respectively, thereby enhancing their external validity [6,18]. Consequently, although adequate for the scope of this single-institution study, the sample size limits the generalisability of our findings to wider populations. Future research with larger and more diverse samples across multiple institutions is recommended to validate and extend our results.

Additionally, this study's reliance on self-reported data may introduce response bias, and future studies could benefit from incorporating objective or qualitative measures to enrich understanding. The use of closed-ended questions, while appropriate for quantitative comparability and brevity, precluded deeper exploration of participants' individual experiences and perspectives; future studies would benefit from incorporating open-ended items or qualitative methods to address this. The study's setting within a single healthcare institution also limits diversity and may affect representativeness, as volunteering opportunities and barriers can differ across regions and institutions. Finally, the long-term impacts of volunteering on interns' professional and personal development remain unexplored and warrant further investigation.

Recommendations for institutions

To enhance volunteer participation among medical interns, institutions should consider addressing common barriers such as time constraints and heavy workloads by integrating volunteering opportunities into academic schedules. Improving communication about available opportunities is crucial, given that 90.0% of non-volunteers cited lack of awareness as a barrier. Additionally, establishing structured programmes with clear guidelines and providing logistical support such as transportation and mentorship could further encourage engagement. Offering incentives such as certificates or career development workshops may also motivate interns, particularly those with career-oriented goals.

Recommendations for further research

Future research should explore the long-term impact of volunteering on medical interns' clinical competency, career trajectories, and personal well-being. Multi-institutional and cross-regional studies would strengthen the generalisability of findings beyond single-institution contexts. Qualitative approaches, including interviews or focus groups, would provide richer insight into the lived experiences and individual perspectives of interns regarding volunteering.

## Conclusions

This study identified the primary motivating factors and barriers influencing volunteering participation among medical interns at Dubai Health. The most frequently cited motivations were serving the community and seeking experiences relevant to career and residency applications, while heavy workload, time constraints, and lack of awareness of available opportunities were the principal barriers among non-volunteers. These findings are consistent with existing literature and highlight specific, addressable gaps in institutional support for intern volunteerism. Improving communication about opportunities and incorporating structured volunteering provisions within intern programmes may meaningfully increase participation. Larger multi-institutional studies are warranted to confirm these findings across broader populations.

## Appendices

Appendix 1

Research Questionnaire

1. Do you agree to participate in the survey?

a. Yes

b. No

2. Did you volunteer previously and/or during this academic year?

a. Yes (Q2 and then we need to check the factors influencing)

b. No (factors preventing them)

3. Type of volunteering activity that you have been involved in: (can select both)

a. Medical

b. Non-medical

4. Which of the following factors influences you to volunteer? (You can select more than one option.)

a. Serving the community

b. Skill enhancement

c. Networking / Building connections and friendships

d. Reducing stress / Enhancing mental health

e. Peers/faculty influence

f. Seeking experiences for residency application/career pursuits (certificates/awards)

g. Availability of sufficient time for volunteering

h. Financial compensation

i. Religious consideration / Good deed

j. Reciprocation / Good Karma

k. Seeking a better understanding of the challenges faced by others

5. Which of the following factors prevents you from volunteering? (You can select more than one option.)

a. Heavy workload / Time constraints

b. Lack of interest

c. Distant location / Transport issues

d. Lack of awareness about volunteering opportunities

e. Physical or mental health issues

f. Financial constraints

g. Lack of legal and policy regulation of volunteering activity

Research Questionnaire adapted with permission from Al Saraidi et al. [4].
